# Feedback, Crosstalk and Competition: Ingredients for Emergent Non-Linear Behaviour in the PI3K/mTOR Signalling Network

**DOI:** 10.3390/ijms22136944

**Published:** 2021-06-28

**Authors:** Milad Ghomlaghi, Anthony Hart, Nhan Hoang, Sungyoung Shin, Lan K. Nguyen

**Affiliations:** 1 Department of Biochemistry and Molecular Biology, School of Biomedical Sciences, Monash University, Clayton, VIC 3800, Australia; milad.ghomlaghi@monash.edu (M.G.); anthony.hart@monash.edu (A.H.); nhoa0001@student.monash.edu (N.H.); sungyoung.shin@monash.edu (S.S.); 2 Biomedicine Discovery Institute, Monash University, Clayton, VIC 3800, Australia

**Keywords:** PI3K/mTOR signalling, feedback loop, crosstalk, nonlinear dynamics, cancer

## Abstract

The PI3K/mTOR signalling pathway plays a central role in the governing of cell growth, survival and metabolism. As such, it must integrate and decode information from both external and internal sources to guide efficient decision-making by the cell. To facilitate this, the pathway has evolved an intricate web of complex regulatory mechanisms and elaborate crosstalk with neighbouring signalling pathways, making it a highly non-linear system. Here, we describe the mechanistic biological details that underpin these regulatory mechanisms, covering a multitude of negative and positive feedback loops, feed-forward loops, competing protein interactions, and crosstalk with major signalling pathways. Further, we highlight the non-linear and dynamic network behaviours that arise from these regulations, uncovered through computational and experimental studies. Given the pivotal role of the PI3K/mTOR network in cellular homeostasis and its frequent dysregulation in pathologies including cancer and diabetes, a coherent and systems-level understanding of the complex regulation and consequential dynamic signalling behaviours within this network is imperative for advancing biology and development of new therapeutic approaches.

## 1. Introduction

The PI3K/mTOR pathway is a critical signalling pathway that is involved in a diverse range of cellular functions, including cell growth, survival, metabolism, protein synthesis and autophagy [1]. The PI3K/mTOR pathway functions to integrate external and internal information to enable cells to make appropriate and efficient decisions regarding growth and proliferation. The pathway is also capable of providing information to, and regulating, other signalling pathways to ensure a cell is unified in its decision making processes [2]. To perform these roles, the PI3K/mTOR pathway has evolved complex feedback mechanisms and has crosstalk with many other signalling pathways, including the cell-cycle regulatory networks, NF-kB signalling, nutrient metabolism, Ras/ERK, and Hippo signalling.

To ensure a cell makes efficient decisions, its signalling networks must be both flexible and robust [3]. Flexibility, in the context of cellular networks, requires that the network has multiple stable output states, and robustness requires that the network be impervious to molecular noise. The manner in which cellular networks achieve this is through the possession of feedback and feed-forward loops that give rise to diverse non-linear dynamics, which facilitate flexibility and robustness. Feedback loops lead to complex behaviours such as bistability, switch-like behaviours, and oscillations [4]. Behaviours like bi-stability and oscillations provide flexibility through the generation of alternate output states, and switch-like behaviour provides robustness through the requirement of a threshold-concentration stimulation before the output changes. On the other hand, feed-forward loops provide mechanisms to speed up the response time [5] and detect fold-change [6] in gene regulatory networks.

Flexibility and robustness are an absolute necessity for cellular networks, but the more flexible and robust the network the more complex it must become. This presents a significant challenge to researchers trying to understand signalling events in a disease context such as cancer. It is becoming increasingly clear that if we want to understand a signalling network to the predictive level, we must uncover their constituent feedback loops and crosstalk, and the consequential network behaviours at the systems level. A prime example of failing to understand a network at the systems level is the underwhelming ability of current PI3K-targeted therapeutics to overcome cancer.

Reflecting its physiological importance, the PI3K/mTOR pathway represents one of the most frequently deregulated pathways in cancer. Genetic aberrations of the pathway components such as PI3K, mTOR, Akt, rapamycin-insensitive companion of mammalian target of rapamycin (RICTOR) and phosphatase and tension homologue deleted on chromosome 10 (PTEN) have been reported in different cancer types [7]. Notably, PIK3CA, the gene encoding the catalytic subunit of PI3K, is among the most commonly mutated genes across many cancer types [8]. Activating mutations in PI3K confer a constitutive ‘on’ signal that decouples external stimulation and growth regulation and drives the dysregulated and unfettered proliferation so integral to oncogenesis. As a result, the PI3K/mTOR pathway is an important target for cancer therapeutics, and a number of targeted drugs directed at this pathway have already been approved for clinical usage [9]. Currently, there are multiple ongoing clinical trials examining the efficacy of PI3K, Akt and mTOR inhibitors either in single or combination treatments with other drugs in different cancer settings (see Supplementary Table S1). Despite the clear appeal of targeting PI3K/mTOR signalling for cancer therapeutics, there are challenges that prevent its full clinical impact. These include off-target toxicity, a lack of mutant specific inhibitors and a lack of predictive biomarkers that can accurately stratify the likelihood of patient response [10], as well as the development of drug resistance [11]. Most of the reasons are, however, underpinned by our lack of a full integrated understanding of the PI3K/mTOR signalling network at the systems level.

In this review, we summarise the complexity in the regulation and dynamic behaviour of the PI3K/mTOR signalling network that emerge from an intricate web of feedback loops, competing protein interactions and pathway crosstalk. We will first provide an overview of signal transduction within the PI3K/mTOR pathway, highlighting important regulatory mechanisms and the non-trivial network behaviours arising from these. We will then describe the crosstalk between the PI3K/mTOR pathway and other signalling pathways and discuss the induction of any feedback this crosstalk might cause. Finally, we end with thoughts on challenges facing the PI3K/mTOR signalling field and provide a perspective on the role of computational modelling in overcoming these.

## 2. Signal Transduction within the PI3K/mTOR Signalling Pathway

**Figure 1:**
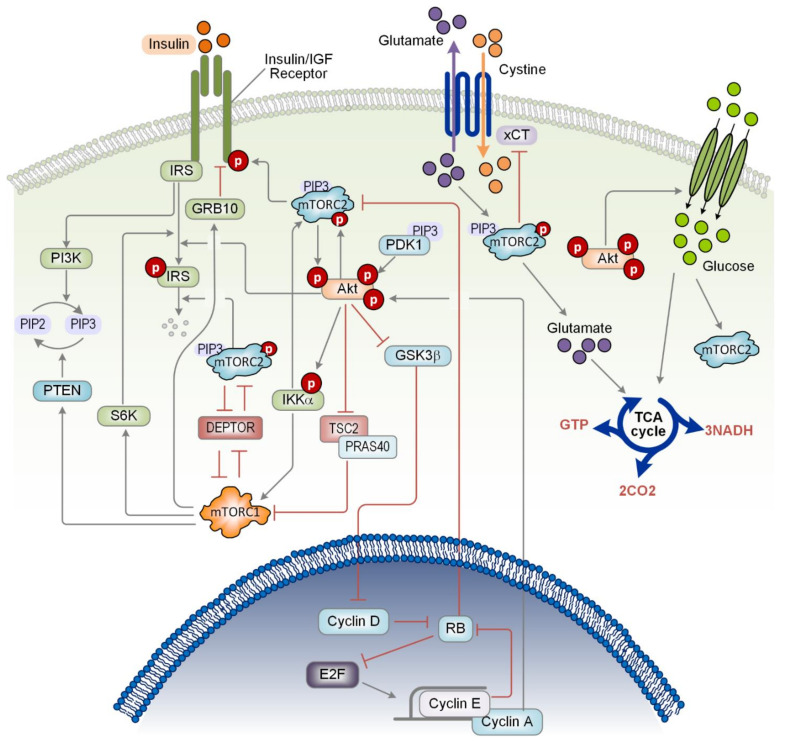
Schematic diagram of the canonical PI3K/mTOR signalling pathway and its interactions with the nutrient sources and cell cycle proteins. For a more detailed description see the main text. Grey arrows indicate positive regulations, while red arrows with blunt ends show negative regulations. Round boxes with P indicate phosphorylation events. The binding of growth factors (e.g., insulin) to IR/IGFR triggers activation of the receptors, promoting the recruitment of IRS and subsequent PI3K activation. PI3K converts PIP_2_ into PIP_3_, which is reversed by PTEN. PIP_3_ recruits mTORC2, PDK1 and Akt to be co-localised at the plasma membrane, leading to the full activation of Akt. Active Akt phosphorylates a plethora of target proteins, including TSC2, GSK3β, IKKα and IRS1/2. Akt also phosphorylates Sin1 and increases mTORC2 activity. IKKα phosphorylates mTOR and stimulates the activation of mTORC1/2, but detailed mechanisms are unclear. DEPTOR displays a mutual inhibition with both mTORC1 and 2. mTORC1 and S6K1 inhibit IR/IGFR through Grb10 and IRS, respectively. mTORC1 upregulates PTEN expression through enhanced translation, while mTORC2 down-regulates IRS1 through ubiquitin mediated degradation. The cyclin A-CDK2 complex phosphorylates and promotes Akt activation. Hyper-phosphorylated Rb localized in the cytoplasm binds to Sin1 and suppresses mTORC2 activity. mTORC2 phosphorylates and down-regulates the functional activity of xCT, which inhibits glutamate efflux and leads to increased TCA cycle activity. Glucose uptake is enhanced by Akt, which leads to activation of mTORC2 through acetyl-CoA-mediated acetylation of RICTOR.

The activation of PI3K usually begins with the activation of an upstream receptor tyrosine kinase (RTK) upon extracellular stimuli, with the insulin receptor (IR) being a canonical activator of PI3K. The binding of insulin to the IR triggers IR dimerization and trans-auto-phosphorylation of IR’s cytosolic domain [12]. The phosphorylation of IR’s cytosolic domain promotes the recruitment of insulin receptor substrate (IRS) through IRS’s pleckstrin homology (PH) domain [13,14]. IRS then acts as an adaptor for the binding of substrates that contain a Src-homology (SH) domain, such as the p85 regulatory subunit of PI3K [15]. In resting cells, p85 stabilises and prevents the catalytic subunit p110 from degradation while simultaneously inhibiting its catalytic activity, keeping PI3K in an inactivated state. However, upon extracellular stimulation and IR activation, the binding of p85 to the IRS and/or IR relieve autoinhibition of PI3K, causing it to accumulate at the cell membrane where the p110 subunit can undertake its function of converting phosphatidylinositol (4,5)-bisphosphate (PIP_2_) into phosphatidylinositol (3,4,5)-bisphosphate (PIP_3_), a second messenger, which is subsequently converted into PtdIns(3,4)P_2_ or PtdIns(4,5)P_2_ by phosphatases, including PTEN, SH2-domain-containing inositol phosphatase 2 (SHP2) and phosphoinositide 3-phosphatases (PI3Ps) [16]. A schematic diagram summarising the canonical PI3K/mTOR signalling interactions is given in Figure 1.

As a membrane-bound second messenger, PIP_3_ is able to recruit PH domain-containing proteins to the plasma membrane, and two major proteins recruited to PIP_3_ are mammalian target of rapamycin complex 2 (mTORC2) and phosphoinositide-dependent protein kinase 1 (PDK1). mTORC2 is recruited to PIP_3_ through the PH domain of the mTOR subunit stress-activated map kinase interacting protein 1 (Sin1). When Sin1 binds PIP_3_, this releases Sin1′s autoinhibition and activates mTOR’s catalytic functions [17,18]. In addition to mTORC2 and PDK1, the protein kinase B (Akt) is also recruited to PIP_3_ via a PH domain. The co-localisation of Akt and activated PDK1 and mTORC2 results in the dual phosphorylation of Akt, where PDK1 phosphorylates Akt at threonine 308 (Thr308), and mTORC2 phosphorylates Akt at serine 473 (Ser473), leading to complete activation of Akt [19,20]. Fully active Akt can then dissociate from PIP_3_ into the cytosol, where it phosphorylates a large range of downstream target proteins.

One of the key downstream proteins governed by Akt activation is mTOR complex 1 (mTORC1). Active Akt phosphorylates tuberous sclerosis complex 2 (TSC2), inactivating it and causing its inhibitory effect on mTORC1 to be shutdown. Newly active mTORC1 can then phosphorylate and activate ribosomal protein S6 kinase (S6K) and inactivates eukaryotic translation initiation factor 4E-binding protein 1 (4E-BP1). The phosphorylation of 4E-BP1 prevents its ability to bind to eukaryotic translation initiation factor 4E (eIF4E), releasing its inhibitory effect on eIF4E and ultimately stimulating the biosynthesis of major classes of macromolecules, including proteins, lipids, and nucleic acids [21]. Other downstream targets of mTORC2 include protein kinase C (PKC), serum/glucocorticoid regulated kinase 1 (SGK1), which regulate cytoskeleton organization and forkhead box ‘other’ (FOXO)-mediated transcription, respectively [22,23,24].

## 3. Complex Regulatory Mechanisms within the PI3K/mTOR Network

Feedback control is fundamental to the robust functioning of cell signalling networks [25]. The existence of multiple positive and negative feedback loops in the PI3K/mTOR pathway plays a critical role in regulating signalling dynamics in response to extra- and intra-cellular perturbations, and their interplay can endow the input-output signalling response with highly nonlinear behaviours. In addition to these explicit feedback mechanisms, subtle protein-protein competitions that emerge from the sharing of subunits between the protein kinase complexes within this pathway can also bring about non-trivial, complex dynamics. In the following sections, we will discuss these regulatory mechanisms in the PI3K/mTOR pathway and the implications for signalling behaviours.

### 3.1. Feedback Mechanisms Mediated by mTOR

Activation of the PI3K/mTOR signalling pathway initiates several mechanisms that are capable of suppressing pathway activity, generating distinct negative feedback mechanisms. Both mTORC1 and S6K can serine-phosphorylate IRS1 (serine 636 and 639) causing it to adopt an inactive form [26,27,28] that subsequently attenuates downstream signalling, including mTORC1/S6K. Further to these well-known negative feedback loops, the PI3K/mTORC1 axis is also under control of another negative feedback mediated by the growth factor receptor bound protein 10 (Grb10), a SH2- and PH-domain containing adaptor protein [29,30,31,32,33]. Upon activation, mTORC1 phosphorylates Grb10 leading to activation and stabilization of Grb10, which inhibits PI3K/mTORC1 signalling by inhibitory binding to tyrosine-phosphorylated IR and IGF receptor [32,33]. Given the similar structure of these feedback loops, why cells have evolved multiple, seemingly redundant, feedback mechanisms to control mTORC1 signalling is intriguing but remains unclear. One possibility is that multiple feedback loops help to provide more robust control in the face of molecular heterogeneity, which may render one of the loops non-functional in specific conditions. It is also possible that coupled negative feedbacks allow enhanced ability to control signalling dynamics, such as tuning different aspects of an oscillatory behaviour [34]. Similar to mTORC1, mTORC2 activity is also associated with the downregulation of IRS1 but does so through ubiquitin-mediated degradation [35]. This is achieved through mTORC2-induced stabilisation of the E3-ligase F-box/WD repeat-containing protein 8 (Fbw8), which allows Fbw8 to translocate into the cytosol and ubiquitinates IRS1. In both cases, the down-regulation of IRS1 activity and expression has a suppressive effect on the downstream proteins [35] (Figure 1).

Recently it has been shown that mTORC1 can promote the activity of the phosphatase PTEN, a key negative PI3K signalling regulator, thereby forming another negative feedback control mechanism [36]. PTEN converts PIP_3_ back into PIP_2_ and can dephosphorylate and activate two negative regulators of mTORC1 activity, TSC1/2 and AMP kinase (AMPK) [37,38]. The study suggests that the expression level of PTEN is positively regulated by mTORC1 via 4E-BP1, where increased eIF4E activity resulting from 4E-BP1 deletion leads to upregulation of PTEN levels [36]. The activation of mTORC1 can thus, via PTEN, negatively regulate upstream components such as PIP_3_ and Akt. A notable implication of this feedback is that targeted inhibition of mTORC1 by pharmacological inhibitors may initially suppress PI3K/mTOR signalling, but the consequential down regulation of PTEN may lead to a rebound in pathway activity over the long-term [36].

Both mTORC1 and AMPK are critical signalling nodes that are responsible for nutrient and energy sensing. AMPK is known to inhibit mTORC1, either directly by phosphorylating RAPTOR at S792 or indirectly by phosphorylating and activating TSC2 at T1387 [39,40]. On the other hand, mTORC1 reciprocally suppresses AMPK activation and signalling by directly phosphorylating the α1 and α2 subunits of AMPK under nutrient stress condition [41]. Moreover, suppression of AMPK activity leads to an increase in phospholipase D (PLD), which in turn inhibits the phosphorylation of AMPK in an mTORC1-dependent manner [42], further supporting their reciprocal regulation [41,42]. Such double-negative feedback connection between AMPK and mTORC1 can give rise to a molecular toggle-switch that locks either of the proteins in the ‘on’ state while keeping the other in the ‘off’ state, which could be exploited for effective therapeutic strategies. For example, co-treatment of the AMPK-activating compound AICAR with rapamycin potently inhibits both mTORC1 and 2, and sensitises cancer cells to rapamycin at clinically tolerated doses [43,44].

PH domain leucine-rich repeat protein phosphatase (PHLPP) is a serine/threonine protein phosphatase with multiple substrates in the PI3K/mTOR pathway [45]. PHLPP dephosphorylates Akt, S6K1, PKC and downregulates their activity [46,47,48,49]. However, pS6K1 in turn induces the protein expression of PHLPP, forming a negative feedback that controls S6K1 activity [50]. Moreover, since GSK3 negatively phosphorylates PHLPP at Serine 847 [51], but Akt inhibits GSK3α and GSKβ through phosphorylation of Serine 9 and Serine 21, Akt thus stabilizes PHLPP expression [52]. Therefore, there are two negative feedback loops regulating S6K1 and Akt activity that are mediated by PHLPP.

In addition to negative feedback regulation, the PI3K/mTOR pathway also contains positive feedback, such as from mTORC2 to IR and insulin-like growth factor receptor (IGF-1R) [53]. mTORC2 is capable of phosphorylating IR and IGF-1R on tyrosine residues 1131/1136 and 1146/1151, respectively, which increases IR/IGF-1R mediated signal transduction. mTORC2 is recruited to IR/IGF-IR through an interaction between the subunit Sin1 and IRS1/2; and it is the kinase activity of mTOR that is responsible for the phosphorylation of the IR/IGF-IR receptors. This positive feedback loop can explain the experimental observation that Sin1 knockdown reduces the activation of IR and PI3K and the abundance of PIP_3_ [35]. The ability of IRS1/2 to recruit mTORC2 is also intriguing as it suggests that PIP_3_ may not be necessarily required for mTORC2 membrane localisation and that IRS1/2 can potentially release Sin1 auto-inhibition and thus activate mTORC2 [17].

Several studies have demonstrated the existence of a negative feedback loop from S6K1 to mTORC2 through its subunits Sin1 and RICTOR, however, this interaction remains controversial [54,55,56]. One route for the negative regulation of mTORC2 by S6K1 is the S6K1-mediated inactivating tyrosine phosphorylation (tyrosine 1135) of RICTOR [55,57]. This phosphorylation does not inhibit the formation of the mTORC2 complex, but RICTOR mutants with a T1135A mutation show increased downstream activation of Akt, suggesting this phosphorylation reduces the kinase activity of mTORC2 [55]. On the other hand, Liu et al. have demonstrated an alternative negative regulation of mTORC2 by S6K1 through S6K1-mediated phosphorylation of Sin1 [54]. The phosphorylation of Sin1 by S6K1 results in impaired mTORC2 integrity, which in turn results in reduced downstream Akt signalling. In contrast, Guang et al. suggest that there is actually no connection between mTORC1/S6K1 and Sin1 phosphorylation [56]. The negative feedback of S6K1 on mTORC2 may be context specific and depend on intermediary proteins or certain expression profiles of the involved proteins. More research will need to be undertaken to clarify the context specificity of this connection.

### 3.2. Feedback Mechanisms Mediated by Akt

It is well established that mTORC2 positively regulates Akt activity by phosphorylating Akt at serine 473 [20,58]. In the opposite direction, Akt has been reported to be capable of phosphorylating the mTORC2 subunit Sin1 at threonine 86 [56]. This phosphorylation relieves Sin1′s auto-inhibitory effect, resulting in increased mTORC2 activity and giving rise to a positive feedback loop between Akt and mTORC2. The mutual activation this feedback loop generates has the potential to produce intricate network response behaviours [59]. One such example is the possibility of bistability wherein initial activation of Akt and mTORC2 results in sustained activity even if the stimulating force is removed. Although the dual phosphorylation of Akt is required for full Akt activation [60], some studies demonstrate that genetic ablation of RICTOR and Sin1, preventing Akt serine 473 phosphorylation, has no effect on a range of Akt substrates, including S6K1 [61,62]. However, other studies suggest that mTORC2-mediated phosphorylation of Akt increases mTORC1 activity and therefore S6K1 activity, once more demonstrating that these feedback loops are likely dependent on specific network conditions [63] (Figure 1).

By combining predictive computational modelling and cell biology, our recent study reveals a new Akt-controlled negative feedback on PI3K-mediated PIP3 that is rapid and powerful [64]. We demonstrate Akt engages this negative feedback by phosphorylating the scaffold proteins IRS 1 and 2, resulting in depletion of IRS1/2 localised at the plasma membrane. This subsequently leads to reduced plasma membrane-associated PI3K and PIP3 synthesis, and ultimately limits Akt activation itself. We identified serines 306 and 577 in IRS2 as the major phosphorylation sites catalysed by Akt that drive the negative feedback. Discovery of this novel negative feedback regulation could explain the limited success of targeting Akt by cancer therapeutics in some contexts [65], as inhibition of Akt is likely to lead to loss of the feedback signal and activation of Akt-independent pro-growth signalling downstream of PIP3.

Akt has a plethora of target downstream substrates, including cytosolic proteins as well as transcriptional factors such as FOXO. Phosphorylation of FOXO by Akt inhibits FOXO and creates a negative feedback loop that suppresses Akt activity [66]. FOXO is involved in apoptosis signalling through the regulation of pro-apoptotic gene expression [67]. Active, unphosphorylated FOXO inhibits protein phosphatase 2A (PP2A), a phosphatase capable of dephosphorylating Akt at serine 473 and threonine 308 [66,68]. Brunet et al. showed that in the presence of growth factor stimulations, Akt inhibits FOXO by phosphorylating it at three sites (threonine 32, serine 253 and serine 315) and in the last two decades, several studies have revealed the broad inhibition of FOXO members by Akt [23,69,70]. The inactivation of FOXO results in the de-inhibition of PP2A, allowing PP2A to regain its suppressive effect on Akt. In addition, FOXO positively regulates the expression of PIK3CA and IR [23,71,72]. Therefore, active Akt negatively regulates the abundance of its upstream components in the PI3K/mTOR pathway, creating another negative feedback mechanism.

### 3.3. Mutual Inhibition Mediated by Protein-Protein Competition

The kinase complexes mTORC1 and mTORC2 form the core of the PI3K/mTOR pathway. While each possesses distinct subunit proteins such as Sin1 and RICTOR for mTORC2, and regulatory-associated protein of mTOR (RAPTOR) and proline-rich AKT substrate of 40 kDa (PRAS40) for mTORC1, they also share common members, notably mTOR-associated protein LST8 homolog (mLST8, also known as GβL) and DEP domain-containing mTOR-interacting protein (DEPTOR). The existence of shared subunit proteins effectively creates competition for these subunits, and so the relationship between mTORC1 and mTORC2 is one of mutual antagonism. Furthermore, a recent study identified a switch mechanism involving reversible ubiquitination of mLST8 that dynamically regulates the relative abundance of the two mTOR complexes [73]. Mechanistically, mLST8 can be ubiquitinated by the E3 ligase TNF receptor-associated factor 2 (TRAF2), preventing mLST8 binding to Sin1 and disrupting mTORC2 assembly [73]. Despite being unable to bind to Sin1, ubiquitinated mLST8 is still able to bind to RAPTOR and thus increases the abundance of mTORC1. However, this regulatory mechanism that promotes mTORC1 abundance can be flipped like a switch. Insulin stimulation activates OTU domain-containing protein 7B (OTUD7B), a protein capable of de-ubiquitinating mLST8 and restoring its affinity for Sin1, which ultimately promotes the formation of mTORC2 [73].

It has been known that the existence of competitive protein interactions coupled with affinity-modulating post-translational modifications can lead to highly non-linear behaviours such as switch-like and biphasic responses [74,75]. To study the emergent behaviours arising from the mLST8-mediated switch, a recent study integrated mechanistic modelling and experimental validation and demonstrated that competition for mLST8 by mTORC1/2 leads to a biphasic dependence of mTORC1 activity on Sin1 [76]. Analysing phosphorylated S6K1 dynamics over a range of concentrations of Sin1 showed that increasing Sin1 below a threshold upregulates mTORC2 formation and promotes mTORC1 activity. However, if Sin1 is increased over the threshold, too much competition exists between mTORC2 and mTORC1 and mTORC1 activity is suppressed instead. From a network point of view, the competition between the two complexes for protein sub-units has a mutual negative effect and this inhibitory mechanism is governed by the regulation of the subunits’ affinities and concentrations.

DEPTOR is another shared subunit of mTORC1 and 2, which acts to negatively regulate them both [77]. Loss of DEPTOR results in enhanced cell growth and survival by upregulating mTOR’s downstream substrates S6K1, Akt and SGK1. On the other hand, both activated mTORC1 and mTORC2 can phosphorylate DEPTOR, which promotes DEPTOR degradation. Thus, mutual inhibition in the form of double-negative feedback exists between DEPTOR and both mTORC1 and 2 [78]. By modelling these mutual antagonistic effects, a systems-based analysis demonstrated that altering the components abundance resulted in the pathway displaying a range of nonlinear behaviours including bi-stability, oscillations, and a mix of both [79]. Their analysis indicates that the mutual negative feedback between DEPTOR and the mTOR complexes drives bistability in the PI3K/mTOR network.

Interestingly, in-silico experiments show that although DEPTOR is considered a negative regulator of mTORC1/2, both low and high levels of DEPTOR have the same effect on Akt activity, Akt hyperactivity. In the case of low DEPTOR abundance, DEPTOR has a greatly reduced inhibitory effect on mTORC1/2 resulting in the complexes having high activity and strong downstream signalling. High DEPTOR expression results in the suppression of the mTOR complexes which has the ultimate effect of suppressing the activity of S6K1. As S6K1 represents a strong negative feedback (discussed above) that shuts down the pathway at the level of IRS, the suppression of S6K1 activity releases its inhibitory effect and promotes Akt activity. Consistent with these findings, both low and high DEPTOR concentrations can be observed in cancer cells [80].

**Figure 2:**
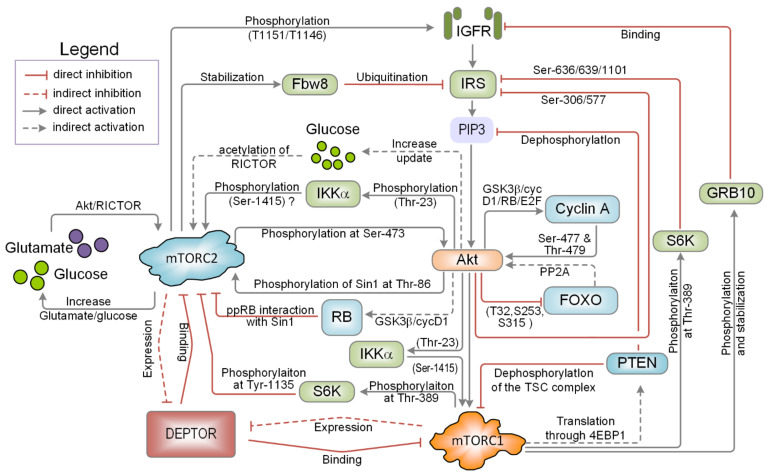
A schematic diagram in the form of flowchart that depicts explicitly multiple feedback and feed-forward loop mechanisms emerging from within the PI3K/mTOR pathway and its crosstalk with other pathways. For each mechanism, the underlying biological details are highlighted. mTORC2 phosphorylates IR and IGF-IR on tyrosine residues 1131/1136 and 1146/1151. mTORC2 stabilises Fbw8 to translocate into the cytosol and ubiquitinate IRS1. Akt enhances glucose uptake, which activates mTORC2 through acetyl-CoA-mediated acetylation of RICTOR. IKKα is phosphorylated at Thr-23 and activated by Akt, which leads to activation of mTORC1 through the phosphorylation at Ser-1415 residue and mTORC2 via an unknown mechanism. mTORC2 phosphorylates Akt at Ser-473 and activated Akt phosphorylates Sin1 at Thr-86 and consequently activates mTORC2. Akt promotes Rb hyper-phosphorylation through inhibition of GSK3β and cyclin D1. Hyper-phosphorylated Rb binds to and inhibits Sin1, leading to inhibition of mTORC2. mTORC1 phosphorylates (at Thr-389) and activates S6K1, which in turn phosphorylates RICTOR at Tyr-1135, inhibiting the mTORC2 activity. S6K also phosphorylates IRS1 at Ser-636/639/1011, causing IRS1 inhibition. DEPTOR blocks mTORC1/2 activation and mTORC1/2 downregulate DEPTOR. mTORC1 phosphorylates and stabilizes Grb10, which is linked to inhibition of IR/IGFR. PTEN dephosphorylates TSC1/2, which blocks mTORC1 activation. mTORC1 increases eIF4E activity, resulting in upregulation of PTEN translation and expression. Akt phosphorylates and inhibits FOXO. Unphosphorylated FOXO inhibits PP2A, which dephosphorylates Akt and blocks its activity. Akt enhances cyclin A expression. The cyclin A2-CDK2 complex phosphorylates Akt at Ser-477 and Thr-479, which stabilises Akt Ser-473 phosphorylation and promotes Akt activity. Glucose uptake is enhanced by Akt, leading to activation of mTORC2 through acetyl-CoA-mediated RICTOR acetylation.

The above discussion highlights that the PI3K/mTOR signalling network possesses many feedback mechanisms, as depicted in Figure 2. These feedback mechanisms produce highly complex and non-linear behaviours that enable a cell to respond to stimuli in an incredibly sophisticated and robust manner. Some of the mechanisms described here seem to only exist under particular network conditions, perhaps mediated by cell lineage or by epigenetic alterations that cause a shift in protein expression. Other mechanisms probably depend on the concentrations of the network’s components, and changing their concentration can alter the feedback strength, ultimately affecting how the network behaves and responds to stimulation. Part of the reason why this network is so complex is that it must integrate and process information coming in from many other signalling pathways and make efficient decisions. In the next section, we will discuss the interplay between the PI3K/mTOR signalling network and other major signalling pathways.

## 4. Crosstalk between PI3K/mTOR and Other Signalling Pathways

Crosstalk between signalling pathways is a vital phenomenon that allows cells to integrate multiple sources of information about the cellular state and make efficient decisions regarding the most beneficial response. When signalling pathways converge on a biological output, such as the stimulation of growth, the topology of the network that controls how the signals are integrated will dictate the types and strengths of response. Pathway crosstalk thus enables a plethora of distinct spatiotemporal response patterns, which may help discriminate between combinations of extra- and intra-cellular cues and result in different cellular decisions [81,82]. Reflecting its pivotal physiological role, the PI3K/mTOR network possesses crosstalk with a large number of signalling pathways, and their inter-dependence will be discussed next.

### 4.1. Complex Crosstalk with the Cell-Cycle Signalling Network

Retinoblastoma protein (Rb) is a tumour suppressor known for its pivotal role in the negative regulation of cell cycle progression. Rb binds to and inhibits the transcriptional factor E2F, causing the cell cycle to be arrested in the G1 phase [83]. Active mitogenic signalling, which includes PI3K/mTOR signalling, stimulates the transcription and accumulation of cyclin D in the cytosol. As cyclin D concentration increases, it binds to its cognate kinase cyclin-dependent kinase 4 and 6 (CDK4/6). The newly formed complex translocates into the nucleus where CDK4/6 phosphorylates Rb, destabilising its ability to bind and repress E2F [84,85]. E2F is then free to transcribe the genes necessary to transition the cell cycle through to S phase (Figure 1).

It has been demonstrated that phosphorylated Rb can become cytosolic, and recently it was shown that phosphorylated Rb can bind to Sin1 and suppress mTORC2 activity [86]. This relationship creates a negative feedback loop between the cell cycle regulatory machinery and the PI3K/mTOR network. The effectiveness of using CDK4/6 inhibitors to prevent cell cycle progression may be limited by this feedback, where reduced phosphorylation of Rb due to CDK4/6 inhibition by the drug agents can result in hyperactive mitogenic signalling through upregulated mTORC2. Experimental evidence regarding this feedback loop however remain inconsistent with one research group showing that hyperphosphorylated Rb suppresses proliferation, but other groups demonstrating that Rb hyperphosphorylation is present in many cancers [87,88].

Cyclin A2 is regulated by E2F and is usually at its highest concentration during S phase. The cognate CDK for cyclin A2 is CDK2, and recently it was demonstrated that the cyclin A2-CDK2 complex can phosphorylate Akt at serine 477 and threonine 479 [89]. These two phosphorylations have the effect of stabilising Akt’s serine 473 phosphorylation and thus upregulate Akt activity. This generates a positive feedback loop between S-phase regulation and the PI3K/mTOR network. It is interesting to note that phosphorylated Rb and the cyclin A-CDK2 complex have opposing effects on Akt. This seems to imply that during the G1 to S-phase transition, Akt signalling is transiently suppressed before being activated once again during DNA synthesis.

### 4.2. Crosstalk with the NF-κB Signalling Pathway

The NF-κB signalling network regulates a diverse set of cellular behaviours, including inflammation, proliferation and cell survival [90]. IKKα is primarily involved in the regulation of the NF-κB signalling network where its activation releases the inhibitory effect of IκB on NF-κB, allowing NF-κB to translocate into the nucleus and regulate transcription. IKKα is known to be activated by the PI3K/mTOR network, specifically through a phosphorylation at threonine 23 by Akt [91]. IKKα is also known to positively regulate mTORC1 and mTORC2, forming a positive feedback loop [92,93]. While it has been shown that IKKα phosphorylates mTOR at serine 1415, leading to the activation of mTORC1, the mechanism through which IKKα activates mTORC2 remains unclear.

Dan et al. demonstrated that the knockdown of IKKα decreased the level of Akt and S6K1 phosphorylation, however, the knockdown did not affect the phosphorylation levels of the Akt substrates TSC2 and PRAS40 [93]. Specifically, IKKα knockdown reduces the phosphorylation of Akt at serine 473, implying that the regulation of Akt might occur through mTORC2. Given that mTORC1 is activated by IKKα, the inhibition of IKKα should de-activate the S6K1-Akt negative feedback loop and stimulate the recovery of Akt phosphorylation. However, the observation that Akt phosphorylation decreases in response to IKKα inhibition suggests that IKKα might also stimulate mTORC2. Overall, the PI3K/mTOR and NF-kB signalling networks positively and mutually reinforce each other, where the stimulation of one pathway leads to both of their signals being amplified (Figure 1).

### 4.3. Crosstalk between mTOR and Nutrient Sources

Feedback nutrient availability is required for biosynthesis, bioenergetics, and redox balance in cells. Glutamate and glucose are two major sources of such nutrients in the majority of cells [94]. xCT is a 12-pass transmembrane that is responsible for the uptake of cysteine, in exchange for glutamate, and is regulated by mTORC2. The phosphorylation of xCT by mTORC2 down-regulates xCT activity and prevents glutamate efflux [95,96]. In the context of cancer, nutrient limitations can drive metabolic adaptations that alter a cell’s source of nutrients. When cancerous cells are glucose starved, they frequently increase glutamate metabolism to maintain the tricarboxylic acid (TCA) cycle to restore energy production [97]. Hyperactivation of xCT was demonstrated to sensitise cancer cells to glucose availability [95]. The increase in xCT activity increases cystine concentration in the cell while inversely reducing the concentration of glutamate [95,98]. This exchange has the effect of increasing a tumour cell’s dependency on glucose uptake and sensitising them to death by glucose starvation, and the inhibition of mTORC2 would act to synergistically increase this sensitisation.

In contrast to the suggestion that mTORC2 is only sensitive to growth-factor stimulation [99], nutrient-dependent mTORC2 activation mechanisms have also been reported. Using an integrated computational-experimental approach, it was demonstrated that amino acids are capable of activating a range of proteins within the PI3K/mTOR network, including PI3K, AMPK, mTORC1 and mTORC2 [100]. Given the positive effect of mTORC2 on glutamate retention in the cell and the positive effect of amino acids on mTORC2, positive feedback exists between glutamate and mTORC2.

Glucose uptake is also affected by mTORC2 and does so through the activation of Akt [101,102,103]. Moreover, glucose release is elevated in RICTOR deficient cells, corroborating the role of mTORC2 in positively regulating glucose uptake [104]. In addition, glucose is also known to activate mTORC2 through acetyl-CoA-mediated acetylation of RICTOR [105]. This provides a second positive feedback loop involving mTORC2 that may contribute to potentially highly non-linear and bi-stable properties of mTORC2 signalling along with the previously discussed mechanisms. Indeed, in a study investigating glioblastoma cells, it was demonstrated that a high level of glucose can convert mTORC2 into a constitutively active kinase, even after the external stimuli is removed [105]. In high glucose conditions, an initial stimulation using EGF led to the sustained activation of mTORC2, which remained active even after EGF was removed from the media. This is further evidence of the bi-stable nature of mTORC2 signalling, where the concentration of glucose can help facilitate the on-state. This also has broad implications for therapies that target proteins such as PI3K and EGFR as the constitutive activation of mTORC2 by high glucose concentrations might severely reduce the therapies effectiveness [105] (Figure 1).

### 4.4. Crosstalk with the Ras/ERK Signalling Pathway

**Figure 3:**
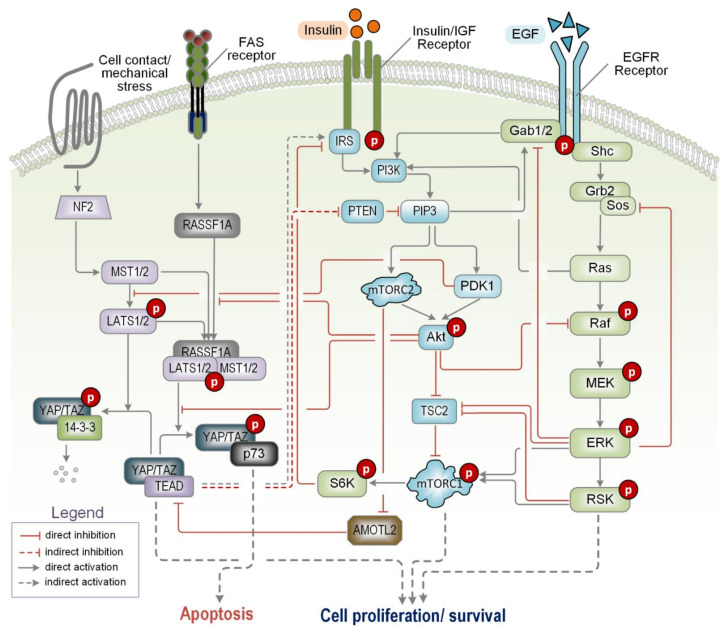
A schematic diagram displaying representative crosstalk interactions between the PI3K/mTOR, Hippo and ERK/MEK signalling pathways. For a more detailed description see the main text. YAP suppresses PTEN translation via the microRNA miR-29. mTORC2 phosphorylates and inhibits AMOTL2, a YAP inhibitor. The phosphorylation of AMOTL2 releases YAP inhibition and promotes YAP-mediated transcription. Akt phosphorylates MST2 at Thr-117 and Thr-384 which blocks MST2 from binding to RASSF1A and inhibits MST2 kinase activity toward LATS. PDK1 promotes the dissociation of MST1/2 from SAV1/WW45 and reduces LATS1 kinase activity. Akt phosphorylates YAP at Ser-127 which retains YAP in the cytoplasm. Akt phosphorylates and inhibits RAF. ERK/RSK phosphorylate and inhibit the TSC complexes, thereby promoting mTORC1 activation. ERK/RSK also directly upregulate mTORC1 through RAPTOR phosphorylation. Tyrosine phosphorylated of Gab1/2 by growth factor stimulation serves as a docking protein for PI3K, which enhances its activation. ERK phosphorylates Gab1/2, triggering its dissociation from the plasma membrane and downregulation of PI3K signalling. RAS interacts with PI3K p110α and promotes PI3K signalling.

Along with the PI3K/mTOR pathway, the mitogenic Ras/ERK (extracellular-signal-regulated kinase) signalling pathway (consisting of the core components Ras, RAF, MEK and ERK) is the cell’s chief regulator of externally stimulated growth, metabolism, and cell survival, and is probably the best described pathway in terms of crosstalk to PI3K/mTOR signalling. There are multiple levels of interplay between the two pathways where they influence each other both negatively and positively, and the coordination of these actions determines complex cell-fate decisions [106] (see Figure 3).

PI3K signalling has long been known as the main effector pathway of Ras where Ras directly interacts with and activate PI3K, forming a major point of crosstalk between the two pathways [107,108]. Importantly, PI3K is required for Ras-induced tumorigenic transformation. Mice with mutations in the PI3K catalytic subunit p110α that render it unable to bind Ras are highly resistant to oncogenic Ras-induced tumorigenesis [109]. Mutant KRAS also has been shown to abnormally induce activation of mTOR complexes which controls protein synthesis and folate cycle [110]. Another well-described crosstalk route is the inhibitory phosphorylation of RAF by Akt on serine 259, where activated Akt can attenuate RAF and downstream signalling [111,112,113]. Conversely, ERK and its kinase substrate p90RSK (90 kDa ribosomal S6 kinase) phosphorylate and suppress the TSC complexes, which negatively regulate mTORC1 activity. Thus, the activity of mTORC1 is upregulated by the Ras/ERK pathway instead. The links mentioned so far form an incoherent feed-forward loop that controls TSC activity: Akt directly inhibits TSC but indirectly promotes it via RAF/ERK. In addition, ERK and RSK can further directly stimulate mTORC1 through the phosphorylation of RAPTOR at multiple serine residues [114,115], creating another incoherent feed-forward loop controlling mTORC1 activity.

Crosstalk also occurs at the level of the plasma membrane. Generation of PIP_3_ by PI3K induces the recruitment of adaptor proteins such as IRS and Grb2-associated binding partner (GAB) to the plasma membrane via their PH domain. Once there, these proteins are phosphorylated on multiple sites by membrane-bound receptors and non-receptor tyrosine kinases, which serve as docking sites to bring additional PI3K molecules to the membrane and enhance the activation of PI3K signalling. This effectively creates a positive feedback loop between PI3K and GAB [116,117]. Importantly, phosphorylated GAB recruits a host of proteins lying upstream of Ras, including Src homology and collagen (Shc), growth factor receptor-bound protein 2 (Grb2) and SH2-domain-containing tyrosine phosphatase 2 (SHP2) that subsequently amplifies Ras/ERK signalling, thereby generating a positive link from the PI3K to Ras/ERK pathway [118]. Interestingly, in the opposite direction, ERK can also phosphorylate GAB1 on serine residues, which has the effect of dissociating GAB1 from the plasma membrane and downregulating PI3K/mTOR signalling, creating an inter-pathway negative feedback loop involving PI3K, GAB, Ras and ERK with a nested PI3K-GAB positive feedback [119]. Moreover, given the negative effect of Akt on RAF discussed above, there further exists a double-negative feedback structure between GAB1, Akt, RAF and ERK that can give rise to toggle switches regulating the pathways’ signalling outputs [120].

ERK also inhibits PI3K signalling via other routes in addition to GAB and TSC. Active ERK and p90RSK phosphorylate and inhibit GSK3 [121], a negative regulator of PTEN. ERK activation therefore alleviates GSK3-mediated PTEN inhibition, subsequently decreasing PIP_3_ levels and PI3K signalling. On the other hand, PDK1 can enhance ERK signalling through phosphorylation of MEK on serine 222 and 226, which is critical for the full activation of MEK [122]. PDK1 also increases MEK/ERK signalling by activation of PKC [123] and PAK1 [124]. Together, these regulatory mechanisms constitute an incredibly intricate web of bidirectional and highly intertwined links between the PI3K/mTOR and Ras/ERK pathways, which are likely to facilitate efficient cell-fate decision making in physiological conditions, but also bring non-trivial and unexpected effects of targeted drugs directed at the nodes of this network [11].

### 4.5. Crosstalk with the Hippo/MST Signalling Pathway

The mammalian Hippo/MST pathway regulates organ size, cell proliferation and cell death. In addition, it has been shown to play a central role in the regulation of cellular homeostasis and is often disrupted in human cancers [125]. In mammals, multiple upstream regulatory proteins such as the scaffolding proteins neurofibromin 2 (NF2) and MOB kinase activator 1 (MOB1) feed into the Hippo pathway and regulate the activity of a core kinase cassette consisting of the serine/threonine kinases STE20-like protein kinase 1/2 (MST1/2) and large tumour suppressor 1/2 (LATS1/2), which in turn control the activity of the co-transcriptional factors Yes-associated protein (YAP) and transcriptional co-activator with PDZ-binding motif (TAZ). Depending on the specific cellular contexts, YAP/TAZ induce expression of both proliferative and apoptotic genes [126,127] (Figure 3).

There are multiple crosstalk between the PI3K/mTOR and Hippo/MST pathways that enable them to mutually regulate each other [128]. One of the early described crosstalk links was the phosphorylation of MST2 by Akt, which inhibits the pro-apoptotic activity of MST2 and its ability to bind the upstream regulator Ras association domain family 1A (RASSF1A), instead favouring MST2 binding to RAF-1 [129,130]. Incorporating these competing protein interactions into a mathematical model that describes crosstalk between Akt, MST2/LATS and the Ras/ERK pathways, we have predicted and confirmed experimentally the presence of steep signalling switches that govern the cell’s decision to switch between proliferation and apoptosis [74,81]. Further network analysis in a subsequent computational study has highlighted diverse and distinct dose-response signalling patterns within this integrated network, which are highly dependent on the specific network conditions [82].

Consistent with the anti-apoptotic role of Akt, PI3K promotes cell survival through the positive regulation of YAP/TAZ, and this has been shown to promote breast cancer progression. The mechanics behind this connection are relatively complex, where PI3K activates PDK1 and Akt, which inhibit LATS and YAP, respectively. Active PDK1 promotes the dissociation of MST1/2 from SAV1/WW45 and reduces LATS1 kinase activity [131,132]. On the other hand, Akt phosphorylates YAP at serine 127, which suppresses YAP-p73 binding and causes YAP to be retained in the cytoplasm where it is unable to regulate transcription [133]. In addition, YAP is also regulated by mTORC2, where mTORC2 phosphorylates and inhibits angiomotin-like protein 2 (AMOTL2), an endogenous inhibitor of YAP [134]. The phosphorylation of AMOTL2 releases YAP inhibition and promotes YAP-mediated transcription. A number of studies have highlighted this link and demonstrated that mTORC2-mediated YAP activation promotes the growth of glioblastoma cells [135,136,137,138,139].

It has been shown that MST1 is capable of forming a complex with and suppressing the activity of Akt, therefore negatively regulating PI3K/mTOR signalling [140]. On the other hand, YAP activity is known to suppress PTEN translation via the microRNA miR-29 [141]. The inhibition of PTEN, a negative regulator of PI3K/mTOR signalling, thus promotes PI3K/mTOR signalling and enhances cell growth. In addition, YAP/TAZ positively regulates IRS2 expression in human hepatocellular carcinoma [142]. Collectively, these discoveries demonstrate a strong interplay between the PI3K/mTOR and Hippo/YAP pathways (see Figure 3) and help explain why YAP hyper-activation is observed in some cancers [143]. The major trend of the connections between these pathways is that of positive feedback loops promoting mutual activation, and their partnership has strong implications for oncogenic progression.

## 5. Concluding Remarks

The PI3k/mTOR signalling pathway is a remarkably complex pathway that contains multiple feedback, feed-forward and competing protein mechanisms; and displays crosstalk with many other signalling pathways. These complex regulatory mechanisms have the potential to induce highly non-linear dynamic signalling behaviours that are too complex for intuitive prediction and reasoning. Computational modelling is thus an instrumental tool with which to investigate such network properties, but the usefulness of any models produced is predicated on accurate and comprehensive biological knowledge [74]. The identification and characterisation of feedback mechanisms are thus critical to the development of predictive models that will enable us to better understand the flow of information through biological networks and design effective therapeutic strategies to overcome complex diseases such as cancer.

One of the themes that has emerged in feedback mechanics is that the cellular context can have a strong influence on which feedback mechanisms are active and are influencing signal transduction. Here, the cellular context refers to any differences between cells and cell types that results in a difference in signal transduction. Examples of these differences include the expression profiles of the relevant proteins and post-translational modifications that alter the protein interactions strengths. Changing the expression level of even a single protein can alter the balance between network states and cause a feedback loop to significantly increase or decrease in strength [76]. A change like this might have the ultimate effect of sensitising the network to external stimulation or inhibition or desensitising the network to the influence of other signalling pathways. It will be of imperative importance for future research to shed light on how the cellular context impacts the way in which information flows through signalling networks and potentially open new avenues for their therapeutic manipulation. Given the relatively straightforward ways in which one could in silico interrogate the effect of altering network states on signalling outputs using computational models (in comparison to experimental methods), modelling and model-based analysis will be critical complementary tools to gain in-depth understanding of context-specific signalling.

The large number of opposing feedback mechanics contained within the PI3K/mTOR network is at first confusing. Why does a network possess multiple feedback mechanisms that seem to counteract each other? One possibility is that this facilitates a very fine-tuned control over signalling outputs. In this scenario, each feedback mechanism influences signalling outputs with varying strengths, giving rise to a range of output levels [144]. Another possibility is that given their central position, PI3K and/or mTOR integrate many sources of cellular information and in order to decide whether to turn growth and survival on or off. Here, the various influences on PI3K/mTOR signalling compete, and if the positive influences outweigh the negative, the switch gets turned on and the cell grows, divides or survives [145]. Nonetheless, understanding the effect that manipulating the various feedback mechanisms has on functional outcomes is also critical to understanding why networks have evolved the way they have. For such tasks, computational modelling and the associated techniques (e.g., perturbation and sensitivity analysis) again offer critical investigative framework [25,82].

The extensive crosstalk exhibited by the PI3K/mTOR and other signalling pathways certainly endows cells with robust abilities for decoding and processing the combinatorial variety of external signals under physiological conditions. Such complex crosstalk, however, also make it hard to predict the possible network-wide effect of cancer therapeutics targeting the pathway’s nodes. For example, pharmacological inhibition of PI3K or Akt may inadvertently activate ERK signalling, allowing tumour cells to evade apoptosis and maintain growth [11]. Moreover, it seems that for a number of PI3K/mTOR’s inter-pathway interactions, if one of the pathways is switched on, the other acts to promote and maintain that on position. This has important implications for PI3K-active cancer, where the inhibition of PI3K/mTOR signalling could be severely compromised if a positively-interacting pathway is allowed to remain active [106,146]. Blocking these pathways, alone or in combination with PI3K/Akt/mTOR inhibition, may have synergistic benefit in overcoming PI3K-driven cancer. Computational modelling has proven to be highly valuable in predicting network-mediated adaptive resistance and identifying effective combination strategies that overcome such resistance and is expected to be of increasing importance for future research [11,147].

Given how frequently the PI3K/mTOR pathway is altered in cancer, it is critical that we continue to investigate and uncover all of the possible feedback mechanisms and pathway interactions. Understanding how the cellular context influences these mechanisms and interactions and how the various PI3K-interacting signalling pathways support and compensate for each other will be equally as important. As research continues, the combination of this knowledge with computational modelling will one day enable us to make incredibly specific and accurate predictions about therapeutic perturbations, down to the individual patient level.

## Supplementary Materials

The following are available online at https://www.mdpi.com/article/10.3390/ijms22136944/s1.
