# Supplementary material for: Feedback, Crosstalk and Competition: Ingredients for Emergent Non-Linear Behaviour in the PI3K/mTOR Signalling Network
Source: Int J Mol Sci. 2021 Jun 28;22(13):6944. doi: 10.3390/ijms22136944 (PMC8267830; doi:10.3390/ijms22136944)
Supplement: Supplementary file 1 [file ijms-22-06944-s001.zip › ijms-1240384-supplementary.pdf]

Table S1. Ongoing cancer clinical trials of drugs targeting the PI3K/mTOR signalling network (source: ClinicalTrials.gov).

Tested regimens	Pathology characteristics	Setting	Phase	Primary outcome measures	No. of patients	Trial identifier
**Gedatolisib / PF-05212384 (dual PI3K/mTOR inhibitor)**						
Gedatolisib + Palbociclib (CDK4/6i)	N/A	Advanced squamous cell lung cancer<br>Advanced pancreatic cancer<br>Advanced head and neck cancer	I	Maximum tolerated dose<br>Incidence of treatment-emergent adverse events	96	NCT03065062
Gedatolisib + Talazoparib (PARP1/2i)	Triple-negative<br>HER2 negative and BRCA1/2 mutation	Advanced or metastatic breast cancer	I and II	Maximum tolerated dose<br>Objective response rate	54	NCT03911973
Gedatolisib + Faslodex (ERi) + Palbociclib (CDK4/6i)	ER positive, HER2 negative	Stage I-IV non-inflammatory invasive breast cancer	Ib	Treatment-related adverse events	18	NCT02626507
**Vistusertib / AZD2014 (mTOR inhibitor)**						
Vistusertib	N/A	Grade II-III intracranial meningioma	II	Progression-free survival	28	NCT03071874
1. Fulvestrant (ERi)<br>2. Vistusertib (continuous schedule) + Fulvestrant (ERi)<br>3. Vistusertib (intermittent schedule) + Fulvestrant (ERi)<br>4. Everolimus (mTORi) + Fulvestrant (ERi)	ER positive, HER2 negative	Advanced or metastatic breast cancer	II	Progression-free survival	333	NCT02216786
1. Vistusertib (continuous schedule) + Olaparib (PARPi)<br>2. Vistusertib (intermittent schedule) + Olaparib (PARPi)<br>3. Capivasertib (AKTi) + Olaparib PARPi)	BRCA1/2 mutation carrier<br>Triple-negative	Endometrial, breast cancer, ovarian, primary peritoneal, or fallopian tube cancer	I and II	Maximum tolerated dose	159	NCT02208375

**Everolimus (mTOR inhibitor)**						
(1. Everolimus<br>2. Placebo	N/A	Invasive unilateral or bilateral breast cancer	III	Benefit from adding everolimus to standard endocrine treatments	1279	NCT01805271
Everolimus + Sorafenib (multikinase inhibitor)	N/A	Thyroid carcinoma	II	Response rate	41	NCT01141309
1. Everolimus followed by STZ-5FU (thymidylate synthetase inhibitor)<br>2. STZ-5FU (thymidylate synthetase inhibitor) followed by Everolimus	N/A	Metastatic or advanced pancreatic neuroendocrine tumours	III	First progression-free survival	141	NCT02246127
**Alpelisib / BYL719 (PI3K inhibitor)**						
1. LSZ102 (ER degrader)<br>2. LSZ102 (ER degrader) + LEE011 (CDK4/6i)<br>3. LSZ102 (ER degrader) + BYL719	ER positive, HER2 negative	Locally recurrent or metastatic breast cancer	I	Incidence of dose limiting toxicities<br>Safety and tolerability	199	NCT02734615
Alpelisib + Letrozole (aromatase inhibitor)	ER positive and/or PR positive and HER2 negative<br>A minimum of 10 patients will need to have a PIK3CA mutation	Stage IV invasive mammary carcinoma	I	Maximum tolerated dose	46	NCT01791478
Alpelisib (250, 300, 350 mg) + Nab-paclitaxel (mitosis inhibitor)	HER2 negative	Locally recurrent or metastatic breast cancer	I and II	Recommended phase II dose of BYL719 + Nab-paclitaxel<br>Overall response rate	44	NCT02379247
**Copanlisib / BAY 80-6946 (PI3K inhibitor)**						
Copanlisib	N/A	Recurrent/refractory solid tumors or lymphoma in pediatric patients	I and II	Maximum tolerated dose<br>Dose-limiting toxicities<br>Treatment-emergent adverse events	142	NCT03458728

				Objective response rate<br>Disease control rate<br>Progression-free survival		
Copanlisib + Nivolumab (PD-1i)	N/A	Relapsed/refractory solid tumors with expansions in mismatch-repair proficient colorectal cancer	I and II	Maximum tolerated dose<br>Objective response rate	54	NCT03711058
1. Copanlisib + Trastuzumab (HER2i) + Pertuzumab (HER2i)<br>2. Trastuzumab (HER2i) + Pertuzumab (HER2i)	HER2 positive<br>PIK3CA or PTEN mutation	Locally advanced or metastatic breast cancer	I and II	Incidence of adverse events and serious adverse events<br>Dose limiting toxicities<br>Progression-free survival	102	NCT04108858
**Inavolisib / GDC-0077 / RG6114 (PI3K inhibitor)**						
1. Inavolisib + Palbociclib (CDK4/6i) + Fulvestrant (ERi)<br>2. Placebo + Palbociclib (CDK4/6i) + Fulvestrant (ERi)	Hormone receptor positive, HER2 negative<br>PIK3CA mutation	Metastatic or locally advanced breast cancer	II and III	Progression-free survival	400	NCT04191499
1. Inavolisib<br>2. Inavolisib + Palbociclib (CDK4/6i) + Letrozole (aromatase inhibitor)<br>3. Inavolisib + Letrozole (aromatase inhibitor)<br>4. Inavolisib + Fulvestrant (ERi)<br>5. Inavolisib + Palbociclib (CDK4/6i) + Fulvestrant (ERi)<br>6. Inavolisib + Palbociclib (CDK4/6i) + Fulvestrant (ERi) + Metformin (energetic stress inducer)<br>7. Inavolisib + Trastuzumab (HER2i) + Pertuzumab (HER2i)	PIK3CA mutation	Locally advanced or metastatic solid tumors	I	Percentage of participants with dose limiting toxicities<br>Recommended phase II dose of Inavolisib<br>Percentage of participants with adverse events and serious adverse events	256	NCT03006172

**Ipatasertib / GDC-0068 (Akt inhibitor)**						
Ipatasertib	N/A	Locally advanced or metastatic solid tumors	I	Maximum/minimum plasma concentration<br>Time to maximum plasma concentration<br>Terminal half-life<br>Apparent clearance<br>Accumulation ratio at steady state	20	NCT04341259
1. Ipatasertib + Fulvestrant (ERi)<br>2. Ipatasertib + Letrozole (aromatase inhibitor)<br>3. Ipatasertib + Fulvestrant (ERi) + Palbociclib (CDK4/6i)	Hormone receptor positive, HER2 negative	Metastatic or locally advanced breast cancer	I	Incidence of treatment-emergent adverse events	60	NCT03959891
1. Ipatasertib + Abiraterone (CYP17i)<br>2. Placebo + Abiraterone (CYP17i)	PTEN loss	Metastatic castrate-resistant prostate cancer	III	Radiographic progression-free survival	1101	NCT03072238
**Capivasertib / AZD5363 (Akt inhibitor)**						
Capivasertib	AKT mutation	Advanced refractory cancers/lymphomas/multiple myeloma	II	Objective response rate	35	NCT04439123
1. Capivasertib + Paclitaxel (ERi)<br>2. Placebo + Paclitaxel (ERi)	Triple-negative	Advanced or metastatic breast cancer	II	Progression-free survival	140	NCT02423603
1. Capivasertib + Paclitaxel (ERi)<br>2. Placebo + Paclitaxel (ERi)	Triple-negative	Locally advanced or metastatic breast cancer	III	Progression-free and overall survival	942	NCT03997123
**MK2206 (Akt inhibitor)**						
MK2206 + Hydroxychloroquine (lysosome inhibitor)	N/A	Advanced solid tumors, melanoma, prostate or kidney cancer	I	Maximum tolerated dose of MK2206<br>Dose-limiting toxicity rate	62	NCT01480154

1. Bicalutamide<br>2. MK2206 + Bicalutamide	N/A	Recurrent prostate carcinoma	II	The proportion of patients with undetectable prostate-specific antigen level	108	NCT01251861
1. Erlotinib (EGFRi)<br>2. AZD6244 (MEK1/2i)<br>3. MK2206<br>4. Lapatinib (HER1/2i)<br>5. Sunitinib (PDGF-R and VEGFR inhibitor)	EGFR mutation<br>KRAS, BRAF, HRAS, or NRAF mutation<br>PIK3CA, AKT, or PTEN mutation<br>KIT or PDGFRA mutation<br>ERBB2 mutation	Advanced non-small cell lung cancer<br>Small cell lung lancer<br>Thymic malignancies	II	Determine the feasibility of the use of tumor molecular profiling and<br>Estimate the response rate of molecular-profile directed treatments targeted therapies	469	NCT01306045
